# The first-in-class alkylating HDAC inhibitor EDO-S101 is highly synergistic with proteasome inhibition against multiple myeloma through activation of multiple pathways

**DOI:** 10.1038/bcj.2017.69

**Published:** 2017-07-28

**Authors:** L Besse, M Kraus, A Besse, J Bader, T Silzle, T Mehrling, C Driessen

**Affiliations:** 1 Department of Oncology and Hematology, Cantonal Hospital St Gallen, St Gallen, Switzerland; 2 Mundipharma-EDO GmbH, Basel, Switzerland

EDO-S101 is a first-in-class alkylating, histone deacetylase inhibitor (HDACi) fusion molecule with dual activity. It structurally combines the strong DNA damaging effect of bendamustine with a fully functional pan-HDAC inhibitor, vorinostat (Supplementary Figure S1) that is intended to simultaneously deliver DNA-damaging activity while inhibiting DNA repair activity, and is currently undergoing phase I clinical testing in hematologic malignancies.^1^ EDO-S101 has shown a strong preclinical activity *in vivo* against multiple myeloma (MM), leukemia and B-cell lymphomas in preclinical models with a toxicity profile similar to bendamustine.^2^ EDO-S101 has remarkable activity *in vivo* in the multi-drug resistant Vk12653 transplant model of relapsed/refractory MM, in which it was the only drug identified with single agent activity.^3^

Bendamustine has substantial activity against B-cell malignancies and vorinostat sensitizes the same type of cancers against alkylators or proteasome inhibitors (PI).^4, 5^ Cytotoxicity of PI in MM relies on excess induction of proteotoxic stress and triggering of the unfolded protein response (UPR)^6^ upon proteasome inhibition, and HDACi synergize with PI by interfering with the α-tubulin-mediated transport of polyubiquitinated proteasome substrates to lysosomal destruction.^7^ Thus, combining EDO-S101 with PI is expected to deliver mechanism-based, highly synergistic cytotoxicity, based on combining proteasome inhibition with histone deacetylase inhibition and alkylating activity in one molecule. We here focus on the preclinical *in vitro* exploration of the potential use of EDO-S101 in combination with PI against MM and B-cell-derived malignancies.

EDO-S101 in combination with PI bortezomib induced the strongest cytotoxic effect compared with vorinostat or bendamustine alone or their combination with bortezomib (Figure 1a). Further, EDO-S101 showed superior cytotoxicity in MM *in vitro* compared to melphalan, cyclophosphamide or bendamustine (Supplementary Figure S2A), showed alike cytotoxicity compared to vorinostat+bendamustine combination (Supplementary Figure S2B) and induced more effective synergistic cytotoxicity in combination with bortezomib and second generation PI carfilzomib, compared to bendamustine or melphalan (Supplementary Figure S2C). Likewise, EDO-S101 induced robust synergistic cytotoxicity in combination with all approved proteasome-inhibiting drugs, as well as PI in clinical development (Supplementary Figure S3, Supplementary Table S1). This synergy was observed already at a 4 μM drug concentration of EDO-S101 and yielded highly significant combination indices for both bortezomib and carfilzomib in a variety of MM cell lines, cell lines from hematologic (mantle cell lymphoma, ABC-type and GC-type diffuse large B-cell lymphoma, acute myeloid leukemia) and non-hematologic malignancies, as well as primary cells from hematologic cancers (Supplementary Figures S4–S6; Supplementary Table S2). The combination between EDO-S101 and carfilzomib was synergistic to overcome bortezomib-resistance, as shown using the AMO-BTZ model previously described^8^ (Supplementary Figure S4A).

The antineoplastic activity of HDAC inhibitors is partly mediated through modulation of nuclear histone acetylation, which results in epigenetic changes, and partly through the modulation of protein acetylation in the cytosol, which functionally controls the activities of several key proteins involved in basic cellular functions as well as oncogenesis.^9^ Acetylation of α-tubulin controls the transport of polyubiquitinated protein to aggresomal proteolysis and is a major molecular mechanism for the cytotoxic synergy between proteasome inhibitors and HDAC inhibitors.^7^ EDO-S101 resulted in superior histone acetylation compared to vorinostat, as revealed by acetylated H3K9, and in particular induced robust acetylation of α-tubulin, also in contrast to vorinostat, suggesting superior inhibition of aggresomal transport of poyubiquitinated protein (Figure 1b). Consistent with this, we observed increased accumulation of polyubiquitinated protein in cells treated with EDO-S101, compared to controls treated with vorinostat or untreated controls. Combined treatment with bortezomib and EDO-S101 led to a maximum increase in cellular polyubiquitinated protein, highlighting the synergy between both pathways. We can exclude a direct effect of EDO-S101 on proteasome activity because EDO-S101 treatment did not change activity-based labeling of active proteasome subunits in treated, viable cells, in contrast to bortezomib (Supplementary Figure S7).

The activation status of the UPR, and in particular the activation levels of IRE1/XBP1 determine proteasome inhibitor sensitivity of MM.^10, 11^ EDO-S101 caused phosphorylation of IRE1, the key activator of the UPR, already 1 h post treatment (Figure 1c). The combination of EDO-S101 with bortezomib resulted in more effective IRE1 activation than either agent alone, as evidenced for example, by the induction of CHOP expression already after 1 h (Figure 1c and Supplementary Figure S8C). IRE1 activation leads to splicing and activation of the XBP1 transcription factor, which significantly increased 4 h post treatment with EDO-S101 or bortezomib treatment alone and further increased upon treatment with the combination of EDO-S101 with bortezomib (Supplementary Figure S8A). By contrast, vorinostat at the same dose did not induce IRE1/XBP1 activation. In summary, treatment with EDO-S101 and bortezomib resulted in robust activation of the UPR, as evidenced by induction of binding immunoglobulin protein (BIP) and protein disulfide-isomerase (PDI) and triggered the ATF4/CHOP signaling pathway that connects proteotoxic stress with the induction of apoptosis (Figure 1c and Supplementary Figure S8B and D). These effects were significantly stronger after combination treatment, compared to either agent alone.

Given the accumulation of polyubiquitinated protein upon simultaneous inhibition of the proteasomal and aggresomal pathways after combination treatment with bortezomib and EDO-S101, we hypothesized that this might activate alternative cellular proteolytic systems, in particular autophagy. Indeed, accumulation of MAP1LC3A/B protein after EDO-S101 treatment, alone and in particular in combination with proteasome inhibition, indicated activation of autophagy upon such dual inhibition of proteasomal and aggresomal protein disposal (Figure 1d).

The alkylating activity of EDO-S101 presumably leads to alkylation of DNA and subsequent double strand breaks, while histone acetylation triggers chromatin de-condensation leading to activation of transcription of cell cycle inhibitors, which both are predicted to result in cell cycle arrest.^12^ EDO-S101 alone caused marked S-phase arrest 8 h post treatment that was further potentiated in combination with bortezomib (Figure 1e). Neither vorinostat nor bendamustine alone or in combination with bortezomib showed significant decrease in cell cycling. Bortezomib-induced cytotoxicity involves upregulation of the CDKN1A (p21) inhibitor of cell cycle via inhibition of p21 degradation.^13^ EDO-S101 alone likewise caused a marked increase in p21 expression already 1 h post treatment, which was further increased during the treatment with a combination of EDO-S101 and bortezomib (Supplementary Figure S9), also in contrast to vorinostat. Conversely, EDO-S101 more effectively reduced CDKN1B (p27) levels alone and in combination with bortezomib, compared to vorinostat (Supplementary Figure S9). Thus EDO-S101 synergizes with proteasome inhibition to induce cell cycle arrest in MM cells.

Overexpression and aberrant activation of c-MYC prevents apoptosis and drives B cell-derived malignancies, including in particular aggressive lymphoma and MM.^14^ EDO-S101 reduced c-MYC expression by 70% already after 4 h, in contrast to vorinostat. The combination of EDO-S101 with bortezomib led to more than 90% reduction in c-MYC expression (Supplementary Figure S10A).

UPR-induced cytotoxicity involves the mitochondrial apoptotic pathway and shifts the balance between pro-apoptotic and anti-apoptotic signals.^15^ EDO-S101 induced expression of pro-apoptotic NOXA (BH3-only BCL2 family member) already 2 h post treatment, while the largest effect was observed 4 h post treatment (Figure 1f and Supplementary Figure S10B). Bortezomib likewise induced NOXA expression, and the combination of EDO-S101 with bortezomib had a more pronounced signaling effect than either agent alone. Consistent with this, EDO-S101 resulted in downregulation of anti-apoptotic BCL2 on protein and transcript levels, and phospho-BCL2 was virtually absent from cells treated with EDO-S101 alone or in combination with bortezomib (Figure 1f and Supplementary Figure S10C). The induction of apoptotic signaling by EDO-S101, alone and in particular in combination with bortezomib, was further supported by an increased ratio of BAX/BCL2 (Supplementary Figure S10D), and was associated with a decrease in the anti-apoptotic effector phospho-MCL1 and, importantly, cleaved PARP (Figure 1f). Strikingly, EDO-S101 alone at 40 μM pulse treatment resulted in 70% apoptotic cells, which increases to 90% when combined with bortezomib 100 nM, while neither equimolar bendamustine nor vorinostat significantly increased the fraction of cells with an apoptotic phenotype by flow cytometry over background levels (20%, Figure 1g).

In summary, EDO-S101 is the first representative of the A-HDAC principle, a new approach in chemotherapy that chemically combines an alkylating agent with a pan-histone deacetylase inhibitor to simultaneously damage DNA and block damage repair. We here show that EDO-S101 has in addition potent ER stress-inducing, HDAC6-inhibiting, immunomodulatory, cell cycle-inhibiting, pro-apoptotic and c-MYC-antagonistic activity, in contrast to vorinostat or bendamustine (Figure 2). This is in line with the superb synergistic cytotoxic activity of EDO-S101 in combination with proteasome inhibitors against MM and a wide variety of B-cell neoplasms *in vitro*, as well as with the activity of EDO-S101 in a predictive *in vivo* model for relapsed/refractory MM. Based on our data, safety and activity of EDO-S101 should be assessed as a potential next-generation alkylating drug with dual, histone-acetylating activity, for multiple myeloma and B-cell malignancies, in particular in combination with proteasome inhibitors.

## Figures and Tables

**Figure 1:**
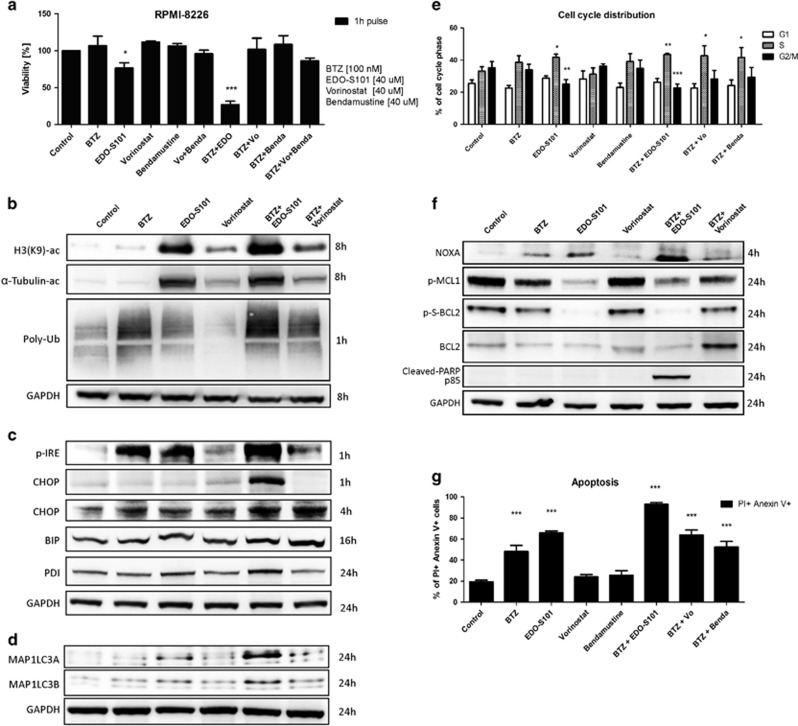
Molecular mechanism of the synergy of EDO-S101 with proteasome inhibitor bortezomib. (**a**) Viability assay comparing cytotoxicity of EDO-S101, vorinostat, bendamustine alone, combination of vorinostat and bendamustine and the combination with bortezomib (BTZ) in RPMI-8226 cell line. Cells were treated with indicated drug concentrations for 1 h and viability was estimated after 48 h. (**b**) Representative western blots in RPMI-8226 cell line depicting inhibition of class I and II histone deacetylases (HDAC) and specifically HDAC6 presented as acetylation of histone H3K9 and α-tubulin, as well as accumulation of polyubiquitinated proteins (poly-Ub). (**c**) Representative western blots depicting induction of ER stress and UPR activation presented by phosphorylation of IRE1 and accumulation of transcription factor CHOP, chaperons BIP and PDI. (**d**) Representative western blots depicting accumulation of autophagosome proteins MAP1LC3A and MAP1LC3B. (**e**) Induction of S-phase arrest observed as an increase of cells distributed in S-phase and decrease of cells in G2/M phase. Cell cycle distribution was evaluated after 8 h. (**f**) Representative western blots depicting NOXA accumulation and downregulation of BCL2, phospho-BCL2 and phospho-MCL1 and cleavage of PARP. (**g**) Functional evaluation of apoptosis by measurement of anexin V/PI positive cells. Apoptosis was evaluated after 24 h. In all experiments, cells were exposed to the indicated drug concentrations for 1 h, followed by removal of the drugs and subsequent incubation in drug-free medium for the indicated time points. Statistically significant differences from untreated controls are marked with asterisks; *P<0.05; **P<0.01; ***P<0.001.

**Figure 2:**
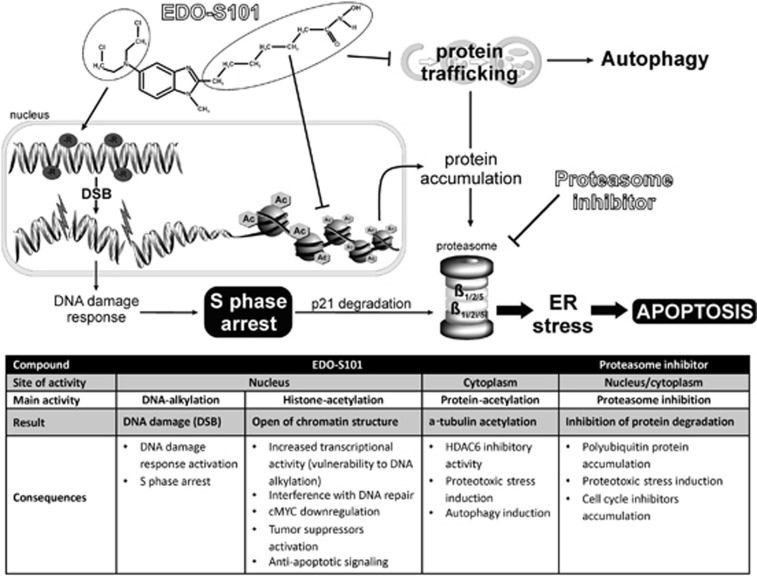
Schematic presentation of EDO-S101 DNA alkylating and HDAC inhibitory role and synergistic effect with bortezomib, and the consequences for the cell. EDO-S101 causes DNA alkylation and histone/protein acetylation due to histone deacetylases (HDAC) inhibitory activity. DNA alkylation leads to the induction of double strand breaks in the DNA, which is potentiated by the vulnerability of the DNA to alkylation due to opened chromatin structure and increased transcriptional activity. The DNA damage and increased transcription of cell cycle inhibitors together with inhibition of cell cycle inhibitors (p21) degradation by bortezomib leads to S-phase arrest. Further, transcriptional activation leads to accumulation of polyubiqitinated proteins dedicated for proteasomal degradation which in turn is blocked by bortezomib. Accumulation of polyubiquitinated proteins causes ER stress which unresolved leads to apoptosis. Intracellular effects are summarized in the table below.
