# Supplementary material for: The first-in-class alkylating HDAC inhibitor EDO-S101 is highly synergistic with proteasome inhibition against multiple myeloma through activation of multiple pathways
Source: Blood Cancer J. 2017 Jul 28;7(7):e589–. doi: 10.1038/bcj.2017.69 (PMC5549260; doi:10.1038/bcj.2017.69)
Supplement: Supplementary Data [file bcj201769x1.pdf]

SUPPLEMENTARY DATA

SUPPLEMENTARY METHODS

Cell lines, primary cells and treatment

All cell lines were obtained from commercial sources (American Type Culture Collection, ATCC; Deutsche Sammlung von Mikroorganismen und Zellkulturen, DSMZ) and were maintained under standard conditions in RPMI-1640 medium (Sigma-Aldrich, MO, USA) supplemented with 10% heat-inactivated fetal bovine serum (FBS), 100 µg/ml streptomycin and 100 U/ml penicillin (Sigma-Aldrich, MO, USA) and were passaged under 20 times and routinely tested for mycoplasma contamination.

Cell lines used:

Multiple myeloma cell lines: AMO-1 and its derivatives resistant to bortezomib (AMO-BTZ) and carfilzomib (AMO-CFZ) (1), INA6, MM1S, MM1R, U266 and RPMI-8226.

Lymphoma cell lines: U937 histiocytic lymphoma, TMD-8 ABC-type B-cell lymphoma, Jeko-1 mantle cell lymphoma, BJAB germinal center/Burkitt lymphoma.

Leukemia cell lines: HL-60 acute promyelocytic leukemia, THP-1 acute monocytic leukemia

Primary cells were obtained from peripheral blood or bone marrow of patients with acute myeloid leukemia (AML), chronic lymphocytic leukemia (CLL) and MM during routine diagnostic procedures after approval by the independent cantonal ethical committee and after obtaining written informed consent form. Where necessary, primary cells were enriched by Ficoll density gradient centrifugation. Primary cell preparations were analyzed microscopically after routine staining and only preparations with > 80% malignant cells were used for experiments described here.

Bortezomib and Carfilzomib were synthetized at the Leiden Institute of Organic Chemistry; other drugs were obtained as described: Vorinostat (S1047; Selleckchem, USA), EDO-S101 (Mundipharma-EDO, Switzerland), Bendamustine (Bendamustine hydrochlorid hydrate; Sigma-Aldrich, MO, USA), Melphalan (Sigma-Aldrich, MO, USA), Cyclophosphamide (Sigma-Aldrich, MO, USA).

Viability assay

Cell viability was determined by MTS tetrazolium compound using CellTiter 96® AQueous One Solution according to manufacturer's recommendations (Promega, WI, USA). For patients' primary cells cell viability was determined by more sensitive CellTiter-Glo Luminescent Cell Viability Assay (Promega, WI, USA).

Gene expression analysis by qPCR

Total RNA was isolated at defined time points of treatment using Trizol (Ambion/ThermoFischer Scientific, MA, USA) and Direct-zol RNA MiniPrep (Zymo Research, CA, USA). 1ug of total RNA was reverse transcribed using High Capacity cDNA Reverse Transcription kit (Applied Biosystems/ThermoFischer Scientific, MA, USA) according to manufacturer's recommendations. Subsequently, 10 ng of cDNA was used into qPCR reactions with 2x LightCycler® 480 SYBR Green I Master Mix (Roche, Switzerland) and the following primers: spliced and unspliced XBP1, ATF4, BIP, CHOP, BAX and BCL2, NOXA, CDKN1A (p21) and CDKN1B (p27), cMYC and GAPDH as a housekeeping gene.

Sequences of primers used for qPCR analysis:

PRIMER	Forward	Reverse
**XBP1 spliced** (2)	5′-CTGAGTCCGAATCAGGTGCAG-3′	5′-ATCCATGGGGAGATGTTCTGG-3′
**XBP1 unspliced** (2)	5′-CAGCACTCAGACTACGTGCA-3′	5′-ATCCATGGGGAGATGTTCTGG-3′
**ATF4** (2)	5′-GTTCTCCAGCGACAAGGCTA-3′	5′-ATCCTGCTTGCTGTTGTTGG-3′
**BIP** (2)	5′-TGTTCAACCAATTATCAGCAAACTC-3′	5′-TTCTGCTGTATCCTCTTCACCAGT-3′
**CHOP** (2)	5′-AGAACCAGGAAACGGAAACAGA-3′	5′-TCTCCTTCATGCGCTGCTTT-3′
**BAX** (3)	5′-TGCTTCAGGGTTTCATCCAG -3′	5′-GGCGGCAATCATCCTCTG -3′
**BCL2** (3)	5′-AGGAAGTGAACATT CGGTGAC -3′	5′-GCTCAGTTCCAGGACCAGGC -3′
**NOXA** (4)	5′-GAGATGCCTGGGAAGAAGG-3′	5′-TTCTGCCGGAAGTTCAGTTT-3′
**CDKN1A (p21)** (5)	5′-TGAGCCGCGACTGTGATG-3′	5′-GTCTCGGTGACAAAGTCGAAG TT-3′
**CDKN1B (p27)** (5)	5′-AGGACACGCATTTGGTGGA-3′	5′-TAGAAGAATCGTCGGTTGCAGGT-3′
**cMYC** (6)	5′-TCAAGAGGCGAACACACAA-3′	5′- GGCCTTTTCATTGTTTTCCA-3′
**GAPDH**	5′-GAAGGTGAAGGTCGGAGT-3′	5′-CATGGGTGGAATCATAATGGAA-3′

Activity based probes labelling

Activity of proteasome subunits after treatment was assessed using the recently developed set of subunit-selective activity based probes (ABP) that differentially visualizes individual activities of β1, β2 and β5 subunits of the constitutive and immunoproteasome (7).

Western blot

Western blot was performed as described previously (8). For the analysis, subsequent antibodies were used: anti-Acetyl-Histone H3 (#9649; Cell Signaling Technology, MA, USA), anti-Acetyl-α-Tubulin (#5335; Cell Signaling Technology, MA, USA), anti-polyUb (PW 8805-0500; Enzo Life Sciences; Lausen, Switzerland), anti-MAP1LC3A (#4599; Cell Signaling Technology, MA, USA), anti-MAP1LC3B (#3868; Cell Signaling Technology, MA, USA), anti-phospho (S724)-ERN1 (IRE1; #ab124945; Abcam, Cambridge, UK), anti-CHOP (Gadd153; #sc-793; Santa Cruz, TX, USA), anti-BIP (GRP78; #610979; BD, New Jersey, USA), anti-PDI (#610946; BD, New Jersey, USA), anti-p21 (#ab109199; Abcam, Cambridge, UK), anti-p27 (#ab32034; Abcam, Cambridge, UK), anti-phospho ERK1/2 (#4370; Cell Signaling Technology,

MA, USA), anti-NOXA (#OP180; Calbiochem/EMD Millipore, MA, USA), anti-phospho (Ser159/Thr163)-MCL1 (#4579; Cell Signaling Technology, MA, USA), anti-BCL2 (#2870; Cell Signaling Technology, MA, USA) anti-phospho (Ser70)-BCL2 (#2827; Cell Signaling Technology, MA, USA), anti-PARP-P85 fragment (#610946; BD, New Jersey, USA), anti-GAPDH POD (Sigma-Aldrich, MO, USA).

Apoptosis evaluation by flow cytometry

RPMI-8226 cells were exposed to the indicated drugs for one hour, followed by removal of the drugs and subsequent incubation in drug-free medium for 24 hours. Cells were stained with the Annexin V/FITC Detection Kit (Biotool, Switzerland) according to the manufacturer's instructions. Cells were then analyzed on FACS Canto II (BD Biosciences, CA, USA), data were analyzed using FACS Diva Software (BD Biosciences, CA, USA).

Cell cycle distribution evaluation by flow cytometry

RPMI-8226 cells were exposed to the indicated drugs for one hour, followed by removal of the drugs and subsequent incubation in drug-free medium for 8 hours. Then, cells were fixed with ice-cold 70% ethanol, incubated with RNAse A (Sigma-Aldrich, MO, USA) for 30 min and propidium iodide for 10 min (Sigma-Aldrich, MO, USA). Cells were analyzed on FACS Canto II and cell cycle distribution was evaluated using FlowJo v10 Software (FlowJo Company, Ashland, OR, USA).

Data evaluation and statistical analysis

Relative quantification of raw qPCR data was performed using 2^{-ddCt} method; data were normalized to housekeeping gene (GAPDH) and to time-point -30 min (-0.5h) of 1h pulse for all the treatments and combinations. The values obtained were then normalized to untreated cells in all the time-points, which served as a baseline. If not specified otherwise, for all experiments are data presented as the mean ± SD of three independent experiments, for flow cytometry data are presented as a median of fluorescence ± SD of three independent experiments. Group comparison for continuous data of qPCR, cell cycle distribution and viability of combination treatment was done with two-way ANOVA with Bonferroni's post-test, for mean comparison of continuous data one-way ANOVA and Bonferroni's Multiple Comparison test was used. P-values < 0.05 were considered as statistically significant in all analyses. Data were statistically analyzed using GraphPad Prism v5. For determination of synergistic cytotoxicity, the combination index (CI) by Chou-Talalay was used, which offers quantitative definition for additive effect (CI = 1), synergism (CI < 1), and antagonism (CI > 1) in drug combinations (9).

SUPPLEMENTARY FIGURES

Supplementary Figure S1: Structure of EDO-S101. EDO-S101 is a fusion molecule of vorinostat (blue) and bendamustine (red).

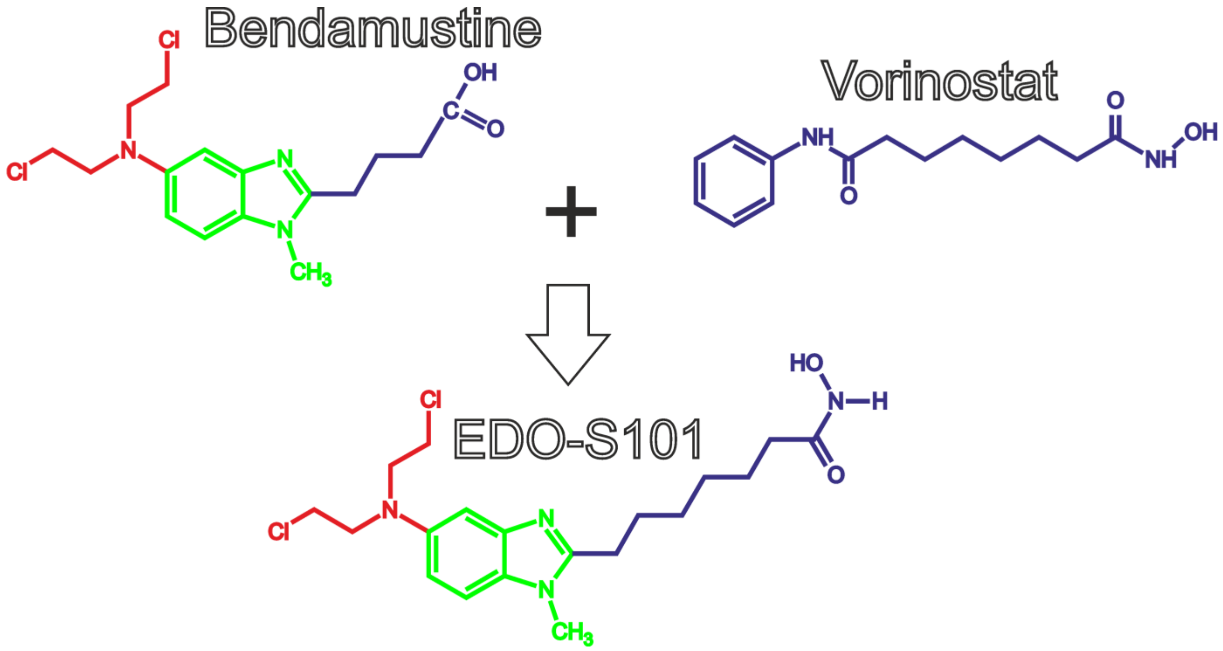

Supplementary Figure S2: Cytotoxic effect of EDO-S101 alone and in combination with proteasome inhibitors. Viability assays in RPMI-8226 cell line comparing EDO-S101 in A) to melphalan, cyclophosphamide, bendamustine, in B) to vorinostat, bendamustine and combination of vorinostat and bendamustine and in C) to combination with proteasome inhibitors bortezomib (BTZ) and carfilzomib (CFZ). Cells were exposed to the indicated drug concentrations for 48h. Statistically significant differences from untreated controls are marked with asterisks; * p<0.05.

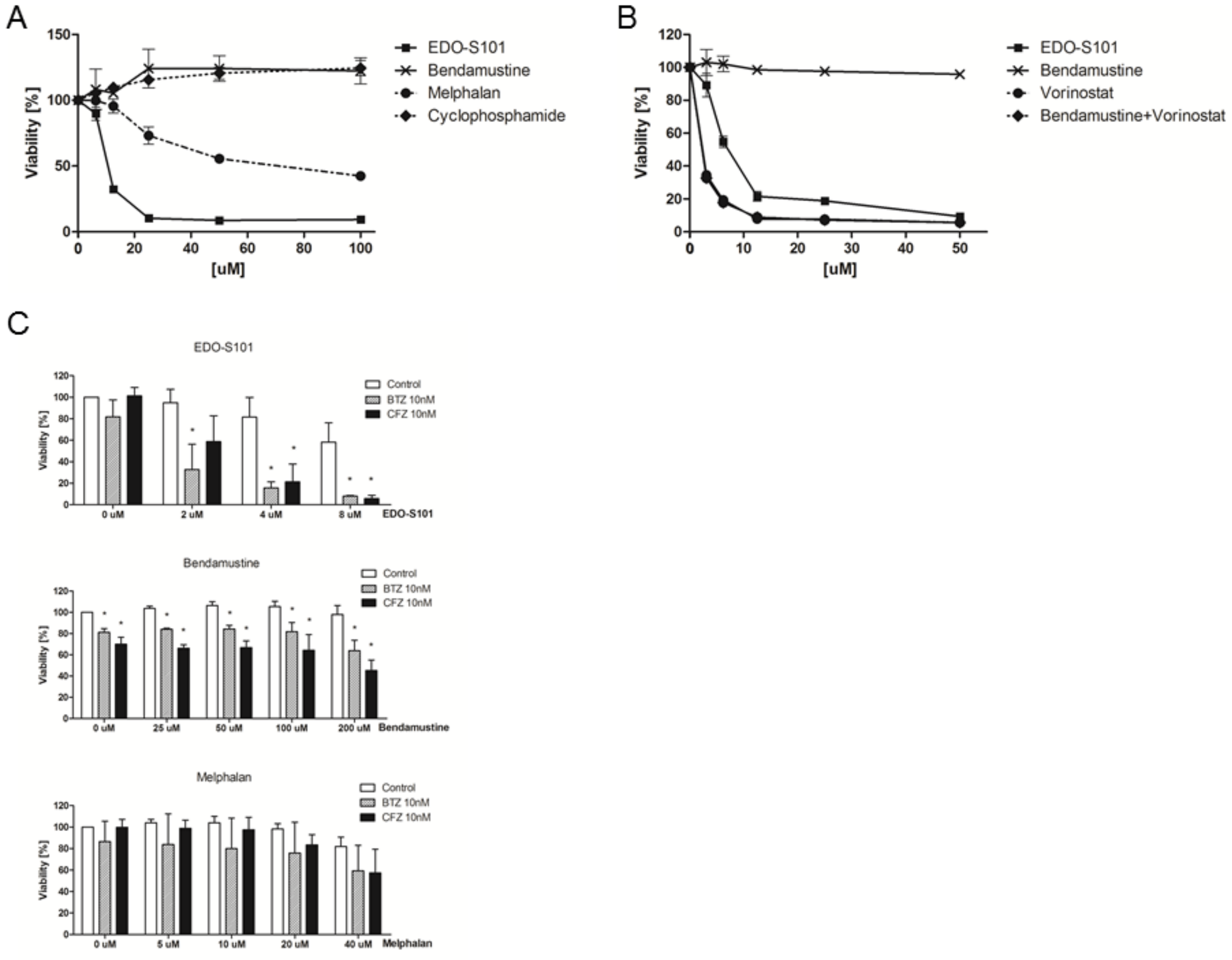

Supplementary Figure S3: Combination of EDO-S101 with different proteasome inhibitors currently approved or advanced clinical development. Viability assay showing cytotoxic effect of EDO-S101 and its synergistic effect in combination with all proteasome inhibitors bortezomib (BTZ), carfilzomib (CFZ), delanzomib (DLZ), oprozomib (OPZ), ixazomib (IXZ), β5 specific inhibitor PR-957, and β2 specific inhibitor Lu-102. AMO-1 multiple myeloma cells were exposed to indicated concentrations of the drugs continuously for 48 hours alone or in combination with 4 µM EDO-S101. IC50 values are presented in Supplementary Table S1.

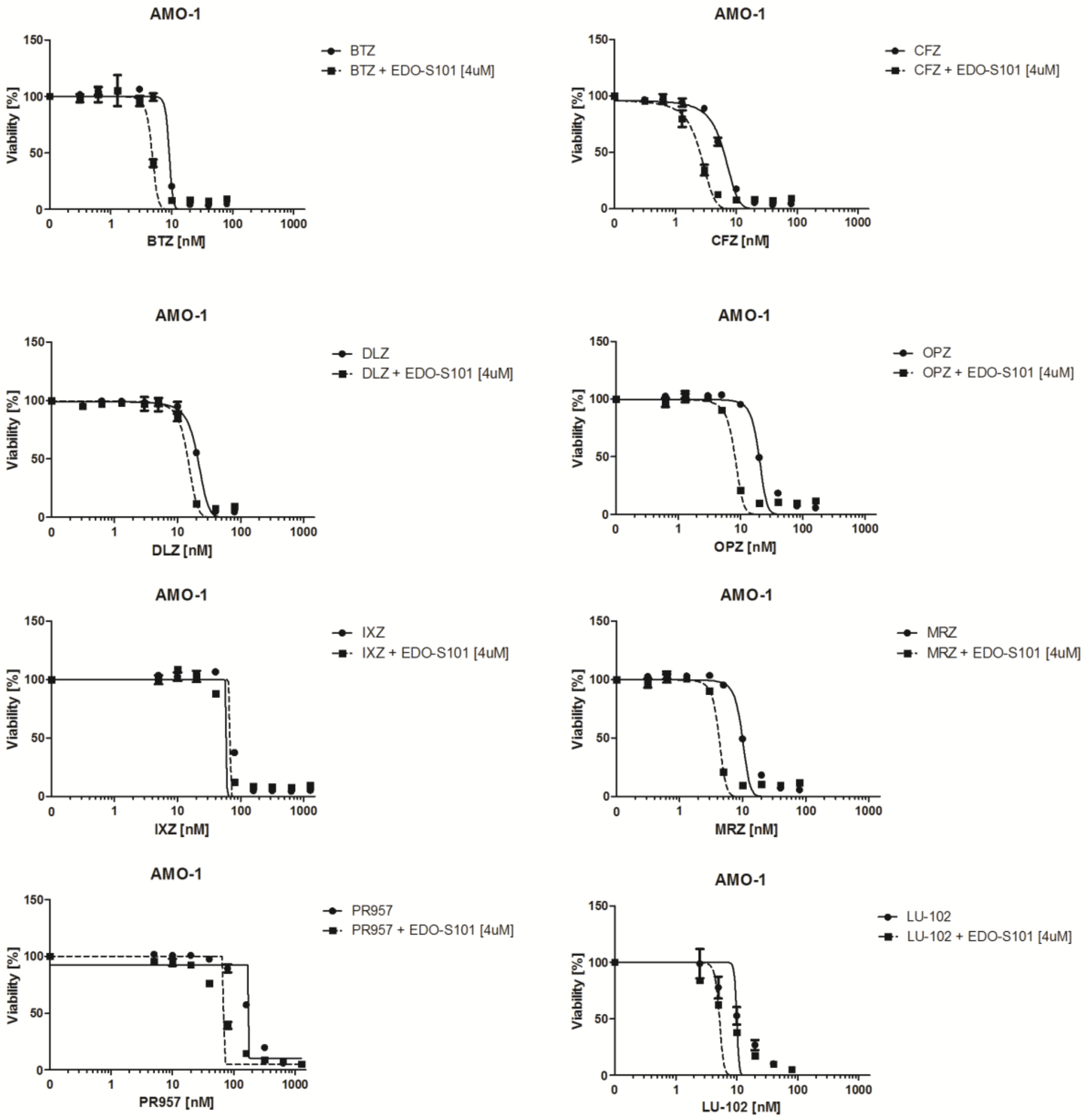

Supplementary Figure S4: Cytotoxic effect of EDO-S101 in combination with proteasome inhibitors in different cell lines of multiple myeloma. Viability assay showing cytotoxic effect of EDO-S101 and its synergistic effect in combination with proteasome inhibitors bortezomib (BTZ) and carfilzomib (CFZ): A) AMO-1 MM cell line and its derivatives resistant to bortezomib (AMO-BTZ) and carfilzomib (AMO-CFZ); B) MM cell lines. In all experiments, cells were treated continuously for 48 hours with indicated concentrations of the drugs. Statistically significant differences from untreated controls are marked with asterisks. * $p<0.05$

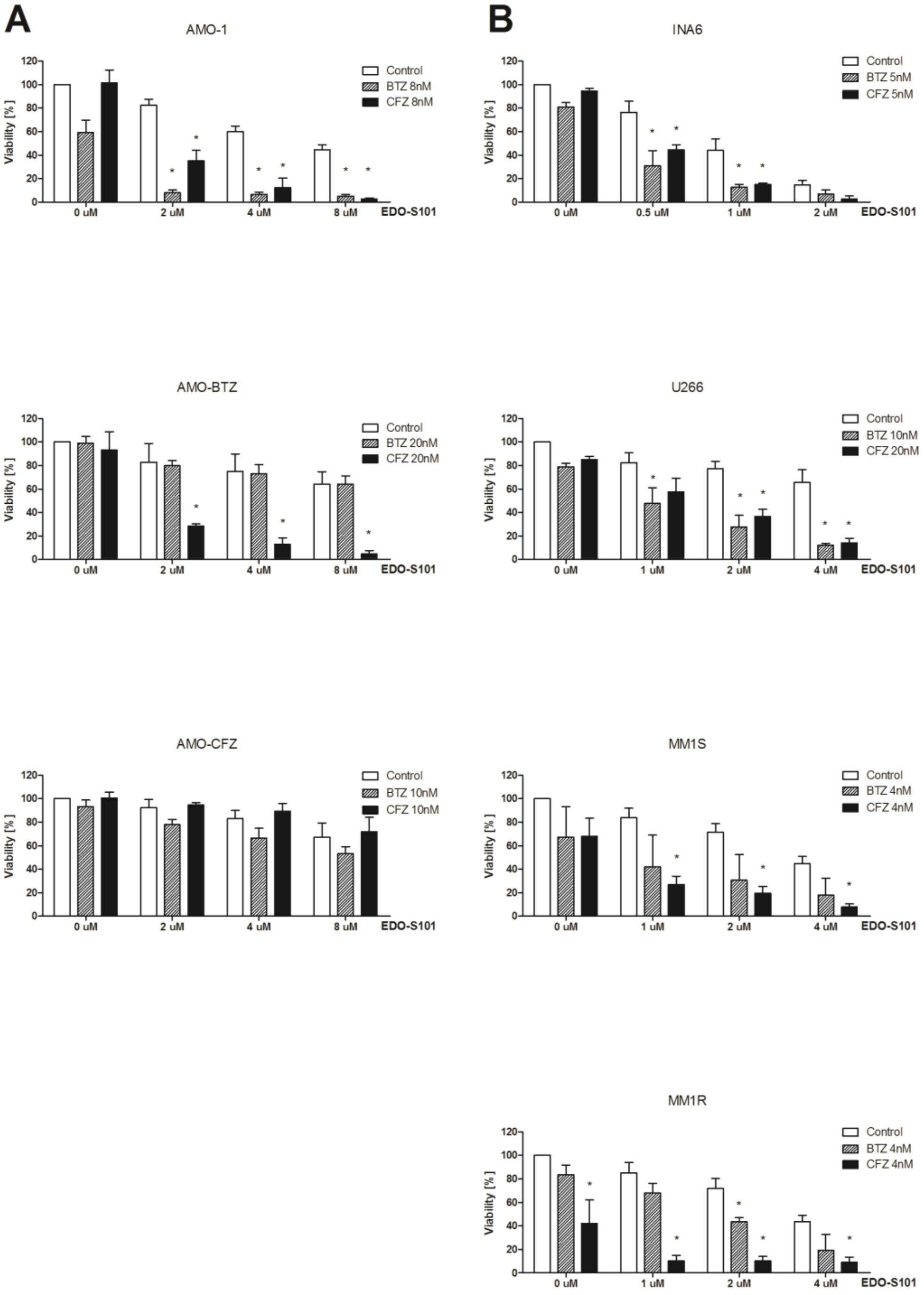

Supplementary Figure S5: Cytotoxic effect of EDO-S101 in combination with proteasome inhibitors in different cell lines of lymphoma, leukemia and triple negative breast cancer. Viability assay showing cytotoxic effect of EDO-S101 and its synergistic effect in combination with proteasome inhibitors bortezomib (BTZ) and carfilzomib (CFZ): A) lymphoma cell lines; B) leukemia cell lines; C) triple negative breast cancer cell lines. In all experiments, cells were treated continuously for 48 hours with indicated concentrations of the drugs. Statistically significant differences from untreated controls are marked with asterisks. * $p<0.05$

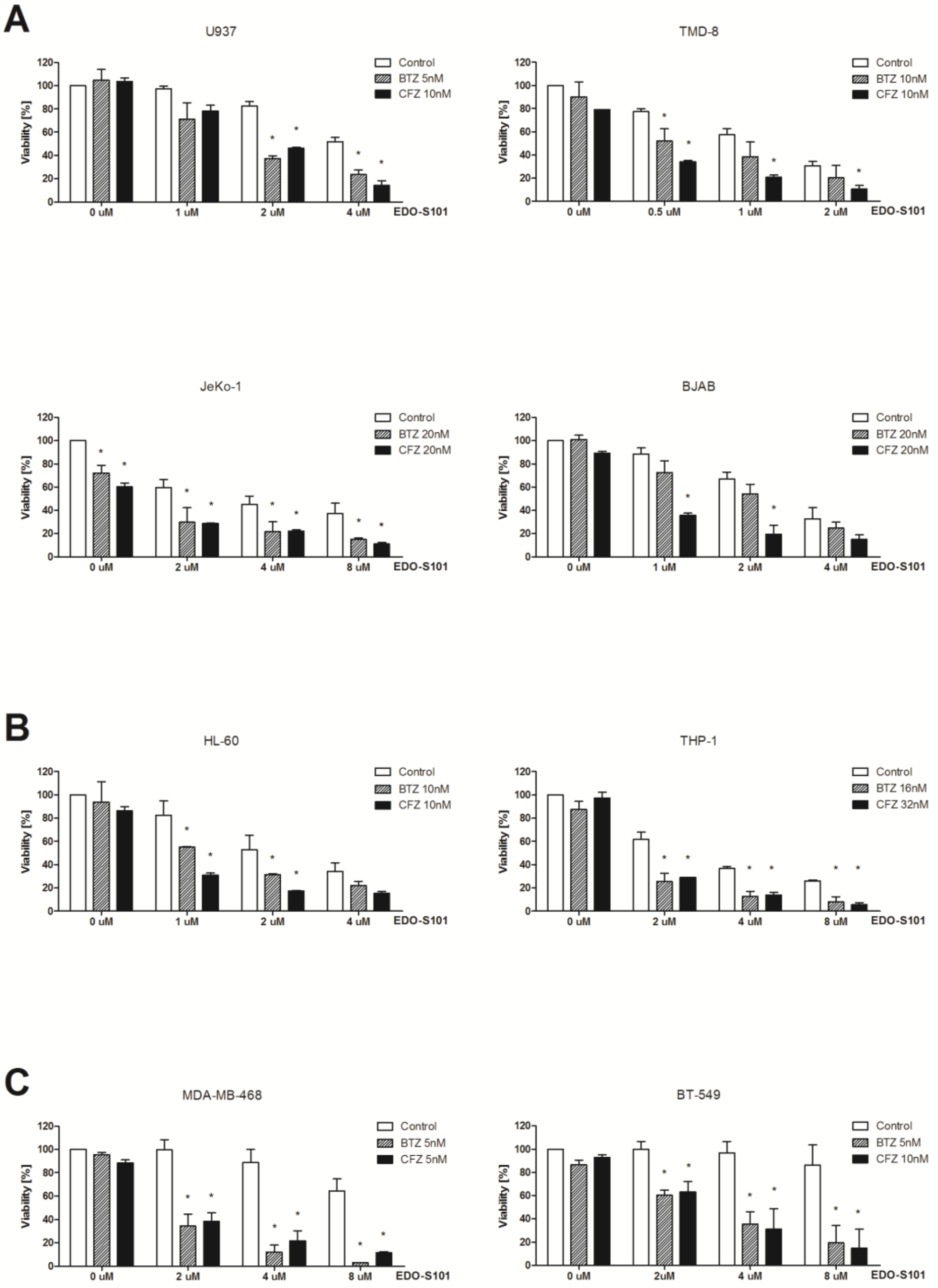

Supplementary Figure S6: Cytotoxic effect of EDO-S101 in combination with proteasome inhibitors (PI) in primary cells of AML, CLL and MM patient resistant to PI. Viability assay showing cytotoxic effect of EDO-S101 and its synergistic effect in combination with proteasome inhibitors bortezomib (BTZ) and carfilzomib (CFZ) in: A) primary AML cells; B) primary CLL cells; C) primary MM cells of PI-resistant patient. In all experiments, cells were treated continuously for 48 hours with indicated concentrations of the drugs. Statistically significant differences from untreated controls are marked with asterisks; * $p<0.05$.

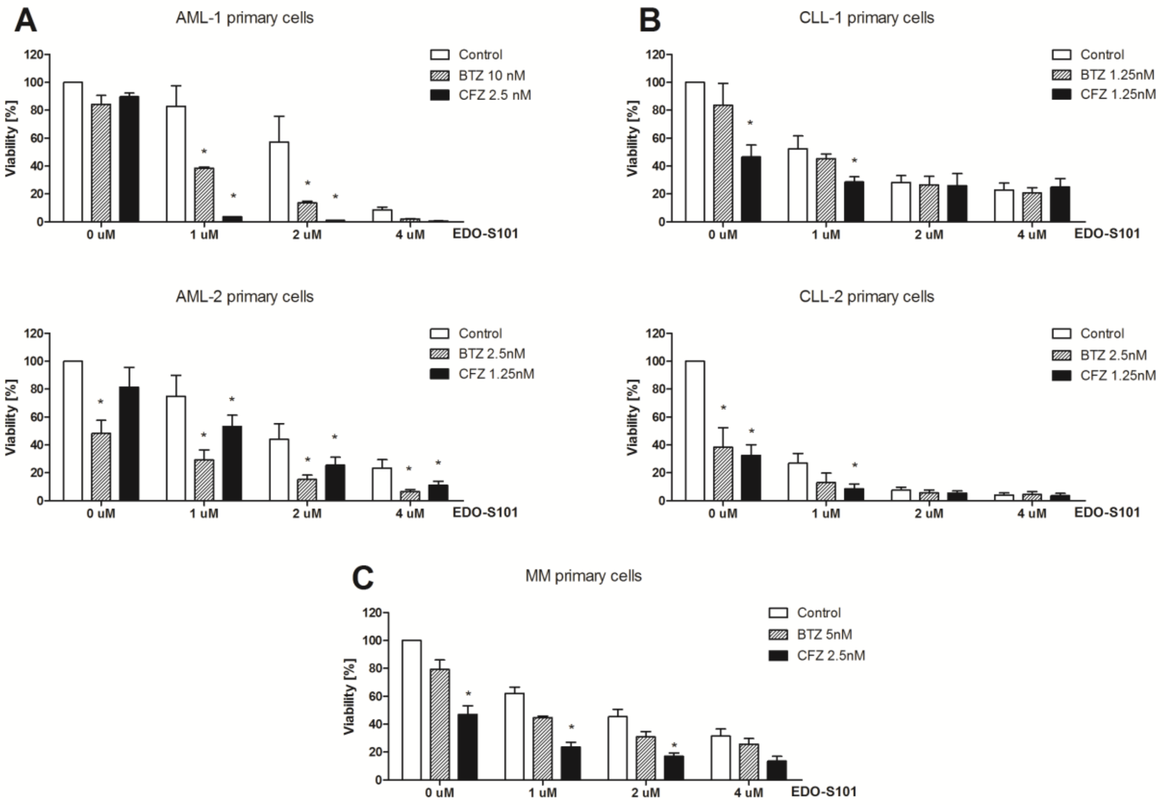

Supplementary Figure S7: Visualization of proteasome activity by active site labelling. A set of activity based probes (ABP) shows activity of constitutive proteasome subunits β1, β2 and β5, and activity of immunoproteasome subunits β1i, β2i and β5i and the decrease of proteasome activity after bortezomib (BTZ) treatment (inhibition β1i and β5/β5i) alone or in combination.

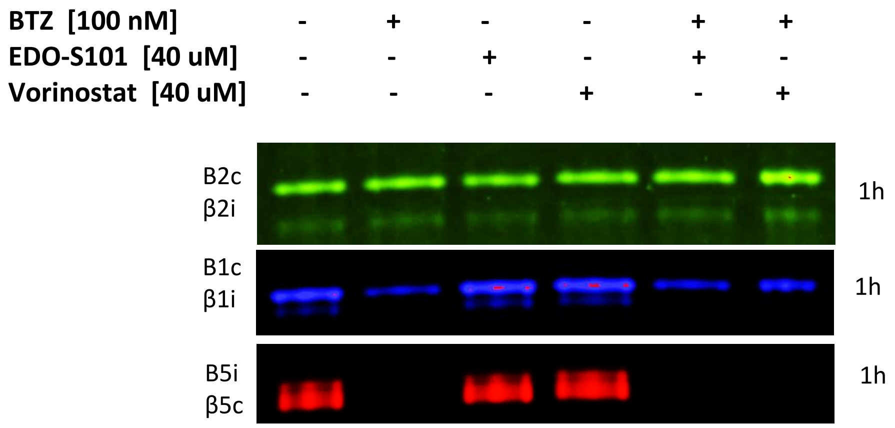

Supplementary Figure S8: ER stress presented by induction of UPR signaling in RPMI-8226 cell line. Induction of A) spliced XBP1 presented as a ratio of spliced versus unspliced XBP1 RNA variants; B) ATF4 C) CHOP and D) BIP expression on RNA levels evaluated by qPCR during indicated time-points. Cells were exposed to the indicated concentrations of the drugs for one hour, followed by removal of the drugs and subsequent incubation in drug-free medium for the indicated time points. Statistically significant differences from untreated controls are marked with asterisks; * $p<0.05$.

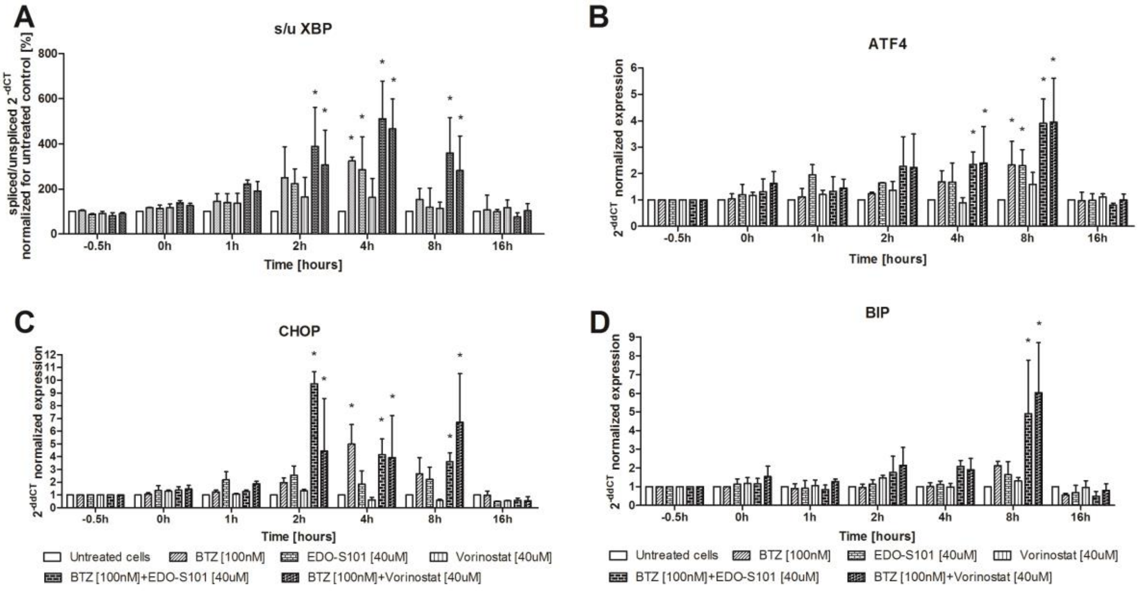

Supplementary Figure S9: Effect of EDO-S101 on cell cycle inhibitors p21 and p27 in RPMI-8226 cell line. A) Downregulation of CDKN1B (p27) and B) upregulation of CDKN1A (p21) expression evaluated on RNA level by qPCR during indicated time-points. C) Representative western blots depicting phosphorylation of ERK1/2, stabilization of p21 and decrease of p27. In all experiments, cells were exposed to the indicated concentrations of the drugs for one hour, followed by removal of the drugs and subsequent incubation in drug-free medium for the indicated time points. Statistically significant differences from untreated controls are marked with asterisks; * $p<0.05$.

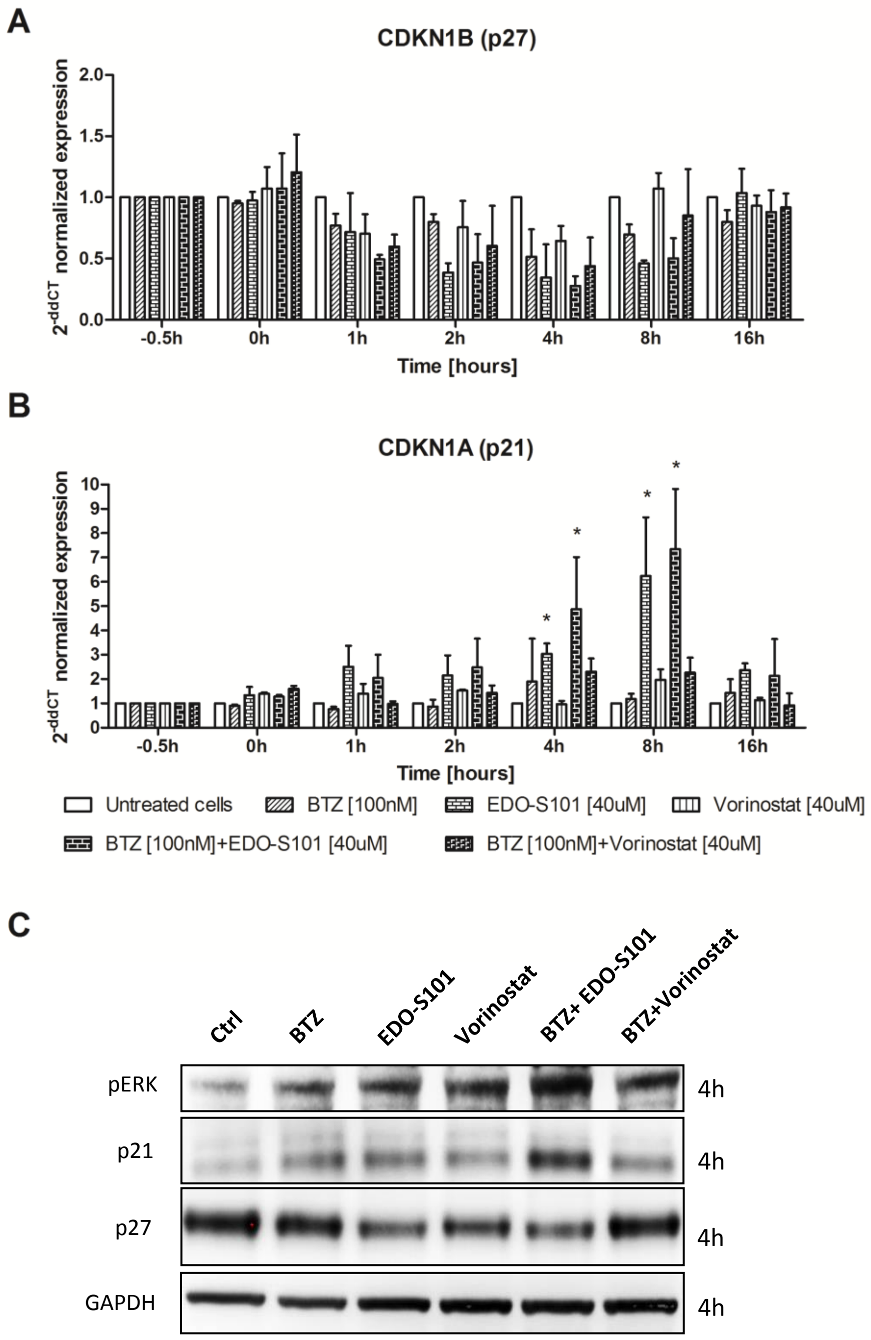

Supplementary Figure S10: Effect of EDO-S101 on the expression of genes involved in the apoptotic signaling in RPMI-8226 cell line. A) Downregulation of cMYC expression, B) upregulation of NOXA expression, C) downregulation of BCL2 expression and D) upregulation of BAX/BCL2 ratio indicating apoptosis evaluated on RNA level by qPCR during indicated time-points. Cells were exposed to the indicated concentrations of the drugs for one hour, followed by removal of the drugs and subsequent incubation in drug-free medium for the indicated time points. Statistically significant differences from untreated controls are marked with asterisks; * $p<0.05$.

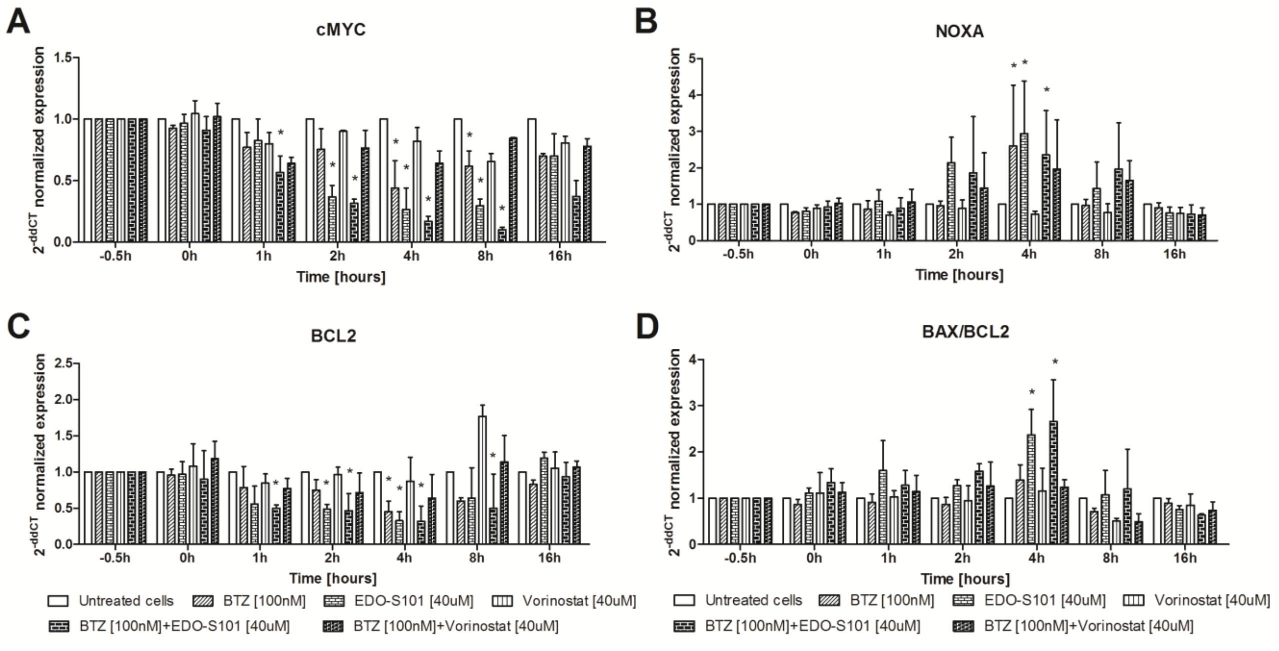

SUPPLEMENTARY TABLES

Supplementary Table S1: IC50 values of proteasome inhibitors alone and in combination with EDO-S101 depicted in Supplementary Figure S2. AMO-1 MM cell line was treated continuously for 48h with different proteasome inhibitors: bortezomib (BTZ), carfilzomib (CFZ), delanzomib (DLZ), oprozomib (OPZ), ixazomib (IXZ), marizomib (MRZ), β5 specific inhibitor PR957 and β2 specific inhibitor LU102 alone or in combination with 4 µM EDO-S101.

IC50 values [nM]	Alone	+ EDO-S101 4 uM
**BTZ**	9.133	4.8
**CFZ**	6.291	2.565
**DLZ**	21.07	14.67
**OPZ**	19.94	8.21
**IXZ**	59.6	68.4
**MRZ**	9.967	4.259
**PR957**	176.7	71.14
**LU102**	10.05	5.291

Supplementary Table S2: Combination indices for EDO-S101 and proteasome inhibitors bortezomib (BTZ) and carfilzomib (CFZ) in different cell lines. Cells were exposed to EDO-S101 and indicated proteasome inhibitors or combination continuously for 48h.

Cell line			Combination index			Combination index
	EDO-S101[uM]	BTZ [nM]	EDO+BTZ	EDO-S101[uM]	CFZ [nM]	EDO+CFZ
**RPMI-8226**	4	10	**0.04**	4	10	**0.07**
**AMO-1**	2	8	**0.01**	8	8	**0.02**
**AMO-BTZ**	2	20	**0.99**	8	20	**0.11**
**AMO-CFZ**	8	10	**0.24**	8	10	**1.17**
**INA-6**	1	5	**0.15**	1	5	**0.22**
**U266**	4	10	**0.11**	4	20	**0.05**
**MM1S**	1	4	**0.18**	1	4	**0.18**
**MM1R**	2	4	**0.33**	1	4	**0.15**
**U937**	4	5	**0.18**	2	10	**0.22**
**TMD-8**	0.5	10	**0.14**	0.5	10	**0.17**
**JeKo-1**	2	20	**0.51**	2	20	**0.27**
**BJAB**	2	20	**0.62**	2	20	**0.09**
**HL-60**	2	10	**0.45**	2	10	**0.36**
**THP-1**	2	16	**0.11**	4	32	**0.14**
**BT-549**	4	5	**0.14**	4	10	**0.05**
**MDA-MB-468**	8	5	**0.13**	8	5	**0.15**

Combination index	
0.0-0.3	high synergy
0.3-0.8	low synergy
0.8-1.0	additivity
more than 1	antagonism

REFERENCES

1. Soriano GP, Besse L, Li N, Kraus M, Besse A, Meeuwenoord N, et al. Proteasome inhibitor-adapted myeloma cells are largely independent from proteasome activity and show complex proteomic changes, in particular in redox and energy metabolism. Leukemia. 2016.
2. Oslowski CM, Urano F. Measuring ER stress and the unfolded protein response using mammalian tissue culture system. Methods Enzymol. 2011;490:71-92.
3. Savli H, Gluzman DF, Sunnetci D, Zavelevich MP, Sklyarenko LM, Nadgornaya VA, et al. Quantitative real time PCR analysis of apoptosis-related gene expression in leukemias in Ukrainian patients. Exp Oncol. 2011;33(2):104-6.
4. Knowlton JJ, Dermody TS, Holm GH. Apoptosis induced by mammalian reovirus is beta interferon (IFN) independent and enhanced by IFN regulatory factor 3- and NF-kappaB-dependent expression of Noxa. J Virol. 2012;86(3):1650-60.
5. Scoumanne A, Cho SJ, Zhang J, Chen X. The cyclin-dependent kinase inhibitor p21 is regulated by RNA-binding protein PCBP4 via mRNA stability. Nucleic Acids Res. 2011;39(1):213-24.
6. Sriskanthadevan S, Jeyaraju DV, Chung TE, Prabha S, Xu W, Skrtic M, et al. AML cells have low spare reserve capacity in their respiratory chain that renders them susceptible to oxidative metabolic stress. Blood. 2015;125(13):2120-30.
7. de Bruin G, Xin BT, Kraus M, van der Stelt M, van der Marel GA, Kisselev AF, et al. A Set of Activity-Based Probes to Visualize Human (Immuno)proteasome Activities. Angew Chem Int Ed Engl. 2016;55(13):4199-203.
8. Kraus M, Bader J, Geurink PP, Weyburne ES, Mirabella AC, Silzle T, et al. The novel beta2-selective proteasome inhibitor LU-102 synergizes with bortezomib and carfilzomib to overcome proteasome inhibitor resistance of myeloma cells. Haematologica. 2015;100(10):1350-60.
9. Chou TC. Drug combination studies and their synergy quantification using the Chou-Talalay method. Cancer Res. 2010;70(2):440-6.
